# Depigmenting Effect of Resveratrol Is Dependent on FOXO3a Activation without SIRT1 Activation

**DOI:** 10.3390/ijms18061213

**Published:** 2017-06-07

**Authors:** Soon-Hyo Kwon, Hye-Ryung Choi, Youn-A Kang, Kyoung-Chan Park

**Affiliations:** College of Medicine, Seoul National University, Seoul National University Bundang Hospital, Gyeonggi 13620, Korea; soonhyo17@hanmail.net (S.-H.K.); hrchoi73@snu.ac.kr (H.-R.C.); k-youn-a@snu.ac.kr (Y.-A.K.)

**Keywords:** extracellular signal-regulate kinase, forkhead box O, melanogenesis, resveratrol, sirtuin-1

## Abstract

Resveratrol exhibits not only anti-melanogenic property by inhibiting microphthalmia-associated transcription factor (MITF), but also anti-aging property by activating sirtuin-1 (SIRT1). In this study, the relationship between depigmenting effect of resveratrol and SIRT1/forkhead box O (FOXO) 3a activation and was investigated. Resveratrol suppressed melanogenesis by the downregulation of MITF and tyrosinase via ERK pathway. Results showed that the expression of both SIRT1 and FOXO3a were increased. It is reported that SIRT1 is critical regulator of FOXO-mediated transcription in response to oxidative stress. However in our study, FOXO3a activation appeared earlier than that of SIRT1. Furthermore, the effect of resveratrol on the levels of MITF and tyrosinase was suppressed when melanocytes were pre-treated with SP600125 (JNK inhibitor). However, pre-treatment with SIRT1 inhibitor (EX527, or sirtinol) did not affect the levels of MITF and tyrosinase. Therefore, resveratrol inhibits melanogenesis through the activation of FOXO3a but not by the activation of SIRT1. Although SIRT1 activation by resveratrol is a well-known mechanism of resveratrol-induced antiaging effects, our study showed that not SIRT1 but FOXO3a activation is involved in depigmenting effects of resveratrol.

## 1. Introduction

The aging process of skin includes pigmentary changes, such as melasma or senile lentigo. Though the mechanism of these phenomena is not clearly understood, much evidence has revealed the association of skin aging and pigmentary changes. Increased levels of melanogenic cytokine or factors were demonstrated in aged skin and chronic ultraviolet (UV)-exposed skin. A significant positive correlation was found between age and interleukin (IL)-1α secretion, which stimulate melanocyte proliferation and tyrosinase activity via hepatocyte growth factor (HGF) and endothelin (ET)-1 secretion [1]. Elevated levels of HGF, keratinocyte growth factor (KGF) and stem cell factor (SCF) were demonstrated in the dermis of senile lentigo [2]. UVB irradiation promotes keratinocytes to secret SCF, basic fibroblast growth factor (bFGF), IL-1, ET-1, inducible nitric oxide synthase, α-melanocyte-stimulating hormone (α-MSH), adrenocorticotropic hormone, and prostaglandin E2 [3,4,5,6,7,8]. Furthermore, basement membrane disruption, a characteristic senile change of skin, was observed in 83 to 95% of the lesional skin in patients with melasma [9].

These findings suggest that depigmenting treatment needs to be accompanied by antiaging treatment. Resveratrol is a hydroxystilbene compound found in grapes, red wine, berries, and other plants and possesses anti-cancer, anti-hyperlipidemia, and anti-aging properties [10]. It has been also reported that resveratrol inhibits the activity of microphthalmia-associated transcription factor (MITF) [11]. Because MITF is a major regulator of melanogenesis [12,13], resveratrol reduced UVB-induced pigment deposition in Yucatan swine [11]. On the other hand, it was reported that tyrosinase inhibition by resveratrol results from a post-transcriptional change of tyrosinase, a rate-limiting step for melnogenesis, but not from MITF alterations [14,15]. One study demonstrated the antioxidant property of resveratrol, which could inhibit tyrosinase [16]. However, we observed that resveratrol at concentrations of 0.1–10 μM did not inhibit tyrosinase directly nor regulate tyrosinase post-transcriptionally [17]. All these findings suggest that depigmenting mechanism of resveratrol still needs further investigation.

Sirtuin-1 (SIRT1) is an NAD+-dependent protein deacetylase, which decreases reactive oxygen species (ROS) levels. It is also involved in variety of cellular processes, such as apoptosis, cell cycle, DNA repair, energy metabolism, and development [18,19,20,21]. SIRT1 modulates two important transcription factors which influence cell survival and death—p53 and forkhead box O (FOXO) [22,23,24]. While there exist p53-independent cell protective pathway, FOXOs were found to be indispensable for SIRT1-dependent cell survival against oxidative stress [25]. Resveratrol activates SIRT1, and thus protects various organs against aging [26,27]. Administration of resveratrol showed beneficial effects against cardiovascular and neurodegenerative diseases [28]. In cultured primary human keratinocytes, resveratrol prevents oxidative stress-induced senescence and proliferative dysfunction by activating the AMP-activated protein kinease (AMPK)-FOXO3a cascade [29]. Involvement of SIRT1 and FOXO in resistance against ROS, insulin signaling, and cellular longevity proposes that modulation of these targets could protect or reverse aged skin or skin pigmentation [30]. However, the relationship between resveratrol-induced depigmenting effects and SIRT1/FOXO3a activation are not clearly demonstrated yet. In this study, we investigated the role of SIRT1 and FOXO3a activation in melanogenesis.

## 2. Results

**Figure 1:**
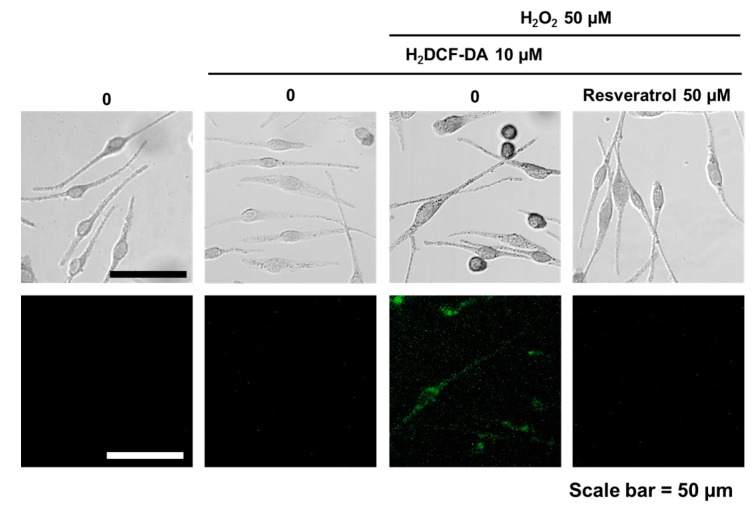
Fluorescent microscopic observation. After resveratrol (50 µM, overnight) treatment, cells then treated by hydrogen peroxide (50 µM, overnight). Fluorescent microscopic examination was done after H_2_DCF-DA (10 µM) staining.

Scavenging activity of resveratrol was tested by DPPH assay. At 0.1 μM, resveratrol exhibited less than 20% of scavenging activity. The antioxidant activity rose dramatically with increasing concentrations of resveratrol (Figure S1). Resveratrol was found to be non-cytotoxic to normal human melanocytes up to 50 μM of concentration (Figure S2). To study the ROS scavenging effects, normal human melanocytes were pretreated with 50 µM of resveratrol for 24 h. Then, H_2_O_2_ (50 µM) was added and examined 24 h later. Results showed that hydrogen peroxide-induced oxidative stress can be ameliorated by 50 µM of resveratrol pre-treatment (Figure 1).

**Figure 2:**
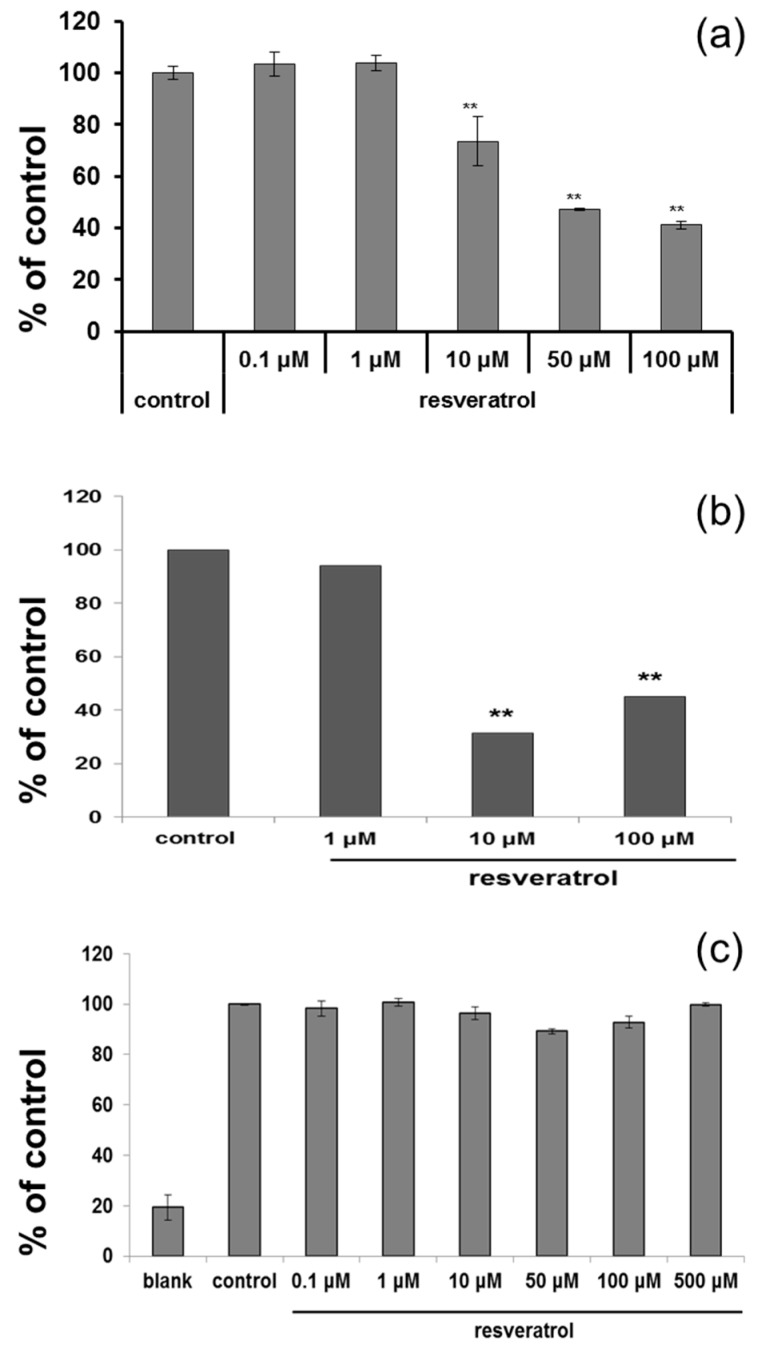
(**a**) ELISA was carried after adding L-DOPA to normal human melanocytes which were treated with resveratrol for three days to measure tyrosinase activity; (**b**) Cells were treated with resveratrol for 72 h and amount of melanin was measured; (**c**) Direct inhibition of tyrosinase by resveratrol was studied using a cell-free system.

Based on cytotoxicity data and ROS scavenging study, 50 µM was selected for further study. After resveratrol treatment, tyrosinase activity was measured. Cultured normal human melanocytes were incubated with resveratrol for three days. After washing, the cells were lysed. Each well of 96-well plate was filled with 90 µL of lysate and 10 µL l-DOPA, then incubated at 37 °C. ELISA was used to measure the absorbance at 475 nm. The activity of tyrosinase was decreased in a dose-dependent manner (Figure 2a). Melanin assay also showed similar results after three days (Figure 2b). However, no direct inhibition of tyrosinase activity was observed at the concentration of up to 500 μM (Figure 2c).

**Figure 3:**
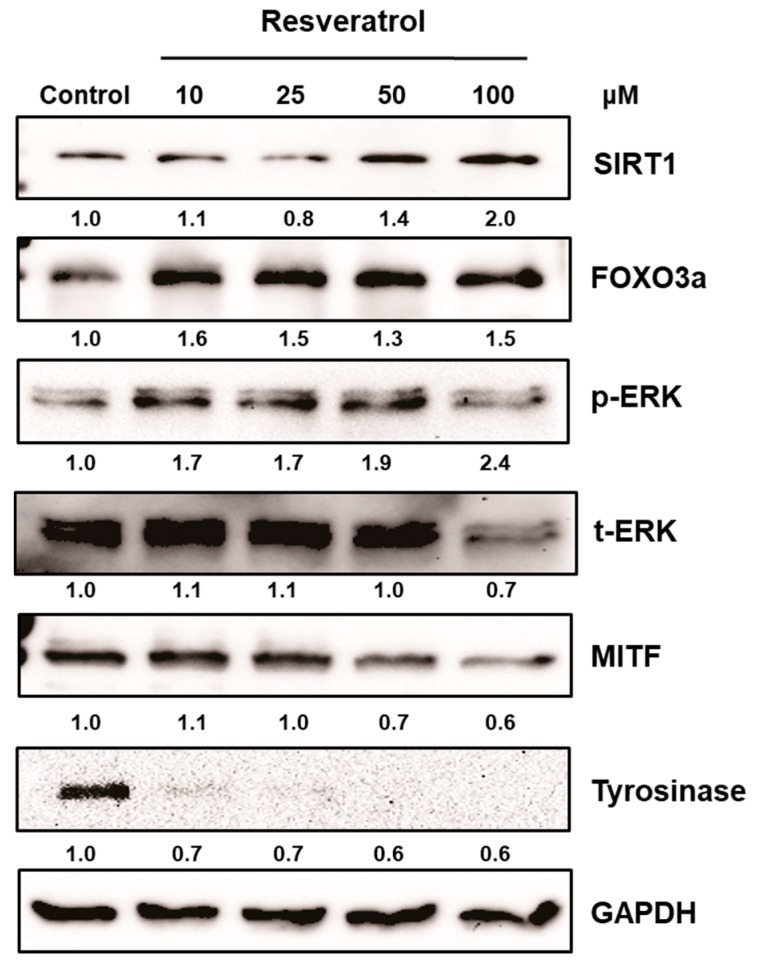
Normal human melanocytes were treated with 10 to 100 μM of resveratrol for 24 h. Then, the levels of ERK, MITF, tyrosinase, SIRT1, and FOXO3a were investigated.

**Figure 4:**
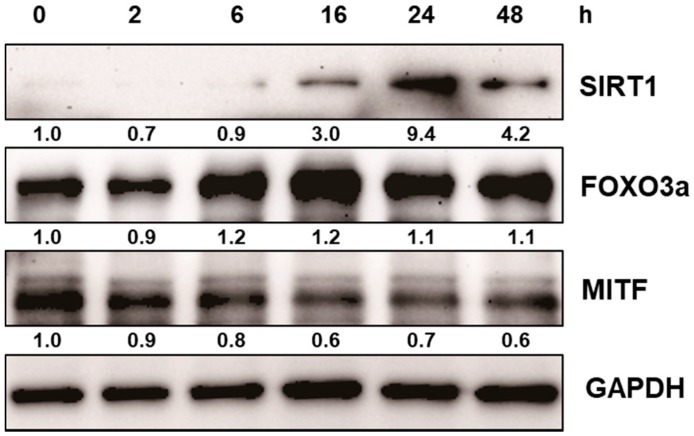
Time-dependent changes of SIRT1 and FOXO3a were investigated after treating normal human melanocytes with 50 μM of resveratrol.

After normal human melanocytes were treated with resveratrol at the concentrations of 10 to 100 μM for 24 h, the levels of extracellular signal-regulate kinase (ERK), MITF, and tyrosinase were investigated via western blot analysis. Resveratrol decreased the levels of tyrosinase and MITF in a dose-dependent manner (Figure 3). Activation of ERK was observed and results verified that the activation of ERK pathway is involved in the inhibition of MITF (Figure S3). Then, the activation of SIRT1 and FOXO3a were investigated. Results revealed that levels of SIRT1 and FOXO3a were also increased in normal human melanocytes after treatment with resveratrol for 24 h (Figure 3). It was reported that FOXO3a is a downstream pathway of SIRT1. Thus, we investigated the time-dependent changes of SIRT1 and FOXO3a after treating cell with 50 μM of resveratrol. Interestingly, our results showed that elevation of FOXO3 appeared earlier than that of SIRT1 (Figure 4).

**Figure 5:**
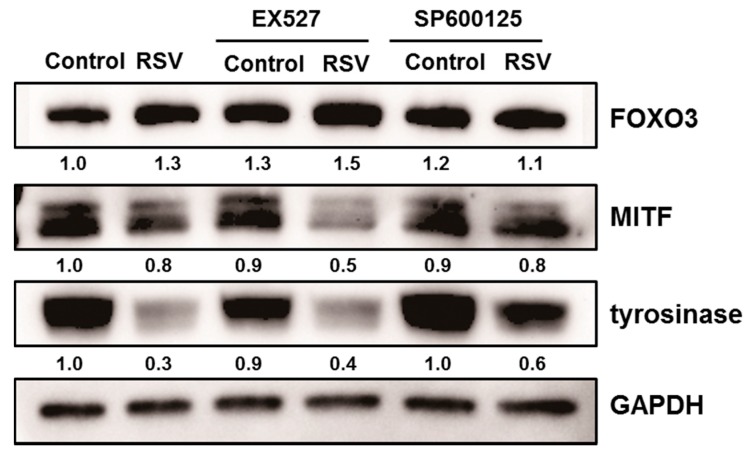
Normal human melanocytes were treated with resveratrol (50 μM) after pre-treated with EX527 (SIRT1 inhibitor) and SP600125 (JNK inhibitor).

**Figure 6:**
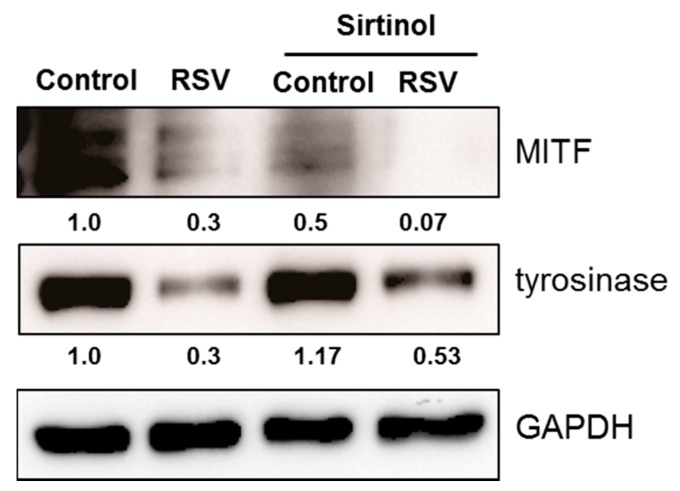
Normal human melanocytes were treated with resveratrol (50 μM) with or without pre-treatment with sirtinol (SIRT1 inhibitor).

In order to analyze the role of SIRT1 or FOXO3a in depigmenting effect of resveratrol, normal human melanocytes were treated with resveratrol after pre-treatment with EX527 (SIRT1 inhibitor) and SP600125 (JNK inhibitor). Resveratrol treatment for 24 h effectively lowered the levels of MITF and tyrosinase (Figure 5). However, pretreatment of EX527 did not change the expressions of MITF and tyrosinase. In contrast, pre-treatment with SP600125 abolished the effect of resveratrol and consequently increased the level of MITF and tyrosinase. These findings suggested that SIRT1 activation is not related with resveratrol induced depigmentation. To confirm the role of SIRT1 on the depigmenting property of resveratrol, cells were pre-treated with sirtinol (SIRT1 inhibitor) to confirm the effect of SIRT1 on MITF and tyrosinase. Results showed that pre-treatment with sirtinol did not affect the effects of resveratrol on MITF and tyrosinase as with EX527 (Figure 6).

**Figure 7:**
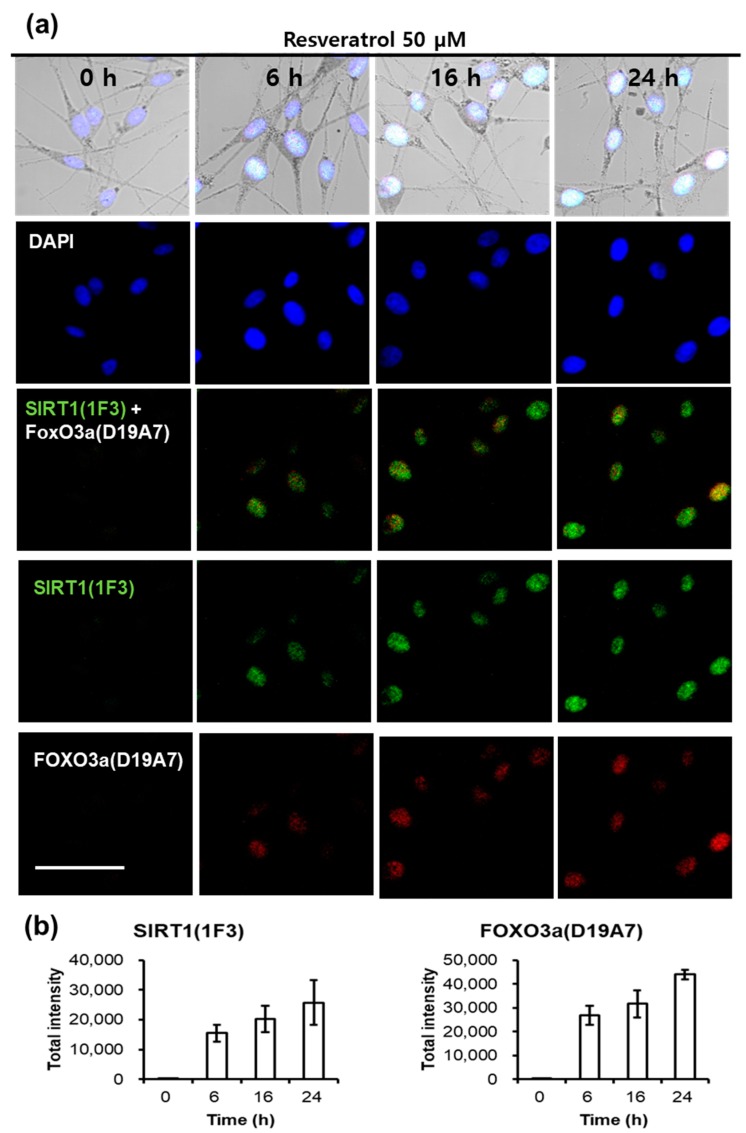
Fluorescent microscopic observation of SIRT1, FOXO3a in resveratrol-treated normal human melanocytes. (**a**) After resveratrol treatment, cells were stained at different time points (0, 6, 16, and 24 h); (**b**) Staining intensity of SIRT1 and FOXO3a after resveratrol treatment at different time points (0, 6, 16, and 24 h). Scale bar = 50 μm.

Fluorescent microscopic examination also showed similar results. The staining intensity of SIRT1 and FOXO3a was observed at a time-dependent manner after treating normal human melanocytes with resveratrol (Figure 7). At 6 h after resveratrol treatment, SIRT1 and FOXO3a appeared simultaneously.

## 3. Discussion

In this study, we found that resveratrol may inhibit tyrosinase through ERK and MITF pathway (Figure S2). In addition, direct inhibitory effect on tyrosinase was not observed [17]. Although there can be some variations, our study show that resveratrol suppressed tyrosinase levels very effectively by 24 h treatment (Figure 3). Though a few studies have suggested that resveratrol might inhibit tyrosinase activity via post-transcriptional regulation of tyrosinase [14], our results showed that resveratrol works through signaling pathways rather than direct inhibition of tyrosinase. Thus, the signaling mechanism of resveratrol in normal human melanocytes was analyzed. Our result also demonstrated that resveratrol could increase SIRT1 (known as longevity protein) and FOXO3a in normal human melanocytes. FOXO3a is a member of FOXO transcription factors which are involved in cellular signaling critical to anti-aging in response to a various stimuli, including insulin, insulin growth factor (IGF), ROS, and cytokines [31]. Recently, FOXO3a was found to be an anti-melanogenic factor that mediates antioxidant-induced depigmentation [31]. In addition, a significant relationship between rs4946936 polymorphism of FOXO3a gene and the occurrence of vitiligo has been demonstrated [32]. Because FOXO3a is modulated by SIRT1, it was hypothesized that resveratrol may increase SIRT1 and the following activation of FOXO3a may be a mechanism of depigmenting effects of resveratrol. In order to study the chronological sequence, we checked the time-dependent changes of SIRT1 and FOXO3a after resveratrol treatment. Unexpectedly, FOXO3a activation appeared earlier than that of SIRT1. Thus, FOXO3a may not be a downstream pathway in resveratrol-induced depigmenting effects.

To investigate the role of SIRT1 and FOXO3a in resveratrol-induced depigmentation, EX527 (SIRT1 inhibitor), and SP600125 (JNK inhibitor) were pre-treated before resveratrol treatment. As expected, the levels of MITF and tyrosinase were decreased after resveratrol treatment. However, the decreased levels of MITF and tyrosinase were restored by the pre-treatment of SP600125. In contrast, pre-treatment with EX527 did not affect the levels of MITF and tyrosinase. It means that FOXO3 has an important role in resveratrol-induced depigmentation process compared to SIRT1 activation. Because there is no specific FOXO3a inhibitor, SP600125 (JNK inhibitor) was used to study the role of FOXO3a in this study (Figure 5). Furthermore, the effects of LY294002 (PI3K inhibitor) were investigated and similar results were obtained (data not shown). Thus, these results suggest that FOXO3a is important mediator in resveratrol mediated depigmentation.

As described, EX527 did not show any effects on resveratrol induced depigmentation. It means that SIRT1 is not involved in resveratrol mediated depigmentation. To confirm the role of SIRT1, sirtinol (another kind of SIRT1 inhibitor) was pre-treated before resveratrol treatment. Results showed that sirtinol also did not reverse the effects of resveratrol on the levels of MITF and tyrosinase either. Therefore, these findings confirm that SIRT1 activation is not involved in resveratrol-induced depigmentation.

Finally, we checked the activation of SIRT1 and FOXO3a by confocal microscopic examination. Results showed that both SIRT1 and FOXO3a began to increase at 6 h after resveratrol treatment. These results are consistent with those by western blotting although the SIRT1 levels were weak by western blotting. Thus, confocal microscopic examination did show again that FOXO3a is not downstream pathway of SIRT1 when melanocytes were treated with resveratrol.

## 4. Materials and Methods

### 4.1. Reagents

SP600125 was obtained from Calbiochem (420119, Darmstadt, Germany). EX527 (203044) and sirtnol (s7942) was purchased from Sigma-Aldrich Co. (St. Louis, MO, USA).

Antibodies that recognize tyrosinase (sc7833) and GAPDH (sc-25778) were purchased from Santa Cruz Biotechnology (Santa Cruz, CA, USA). Antibodies recognize total (phosphorylated and non-phosphorylated) ERK1/2 (9102), phospho-specific ERK1/2 (Thr202/Tyr204, 9101S), FoxO3a (12829S), and SirT1 (8469s) were purchased from Cell Signaling Technology (Danvers, MA, USA). Antibody to MITF (MS-771-PI) was purchased from ThermoScientific Nunc (Rochester, NY, USA). For confocal staining, Alexa Fluo488 chicken anti-rabbit IgG (H + L) (A21441, Molecular Probes, Eugene, OR, USA), and Alexa Fluo555 donkey anti-goat IgG (H + L) (A21432, Molecular Probes, Eugene, OR, USA) were obtained from Invitrogen (Carlsbad, CA, USA).

### 4.2. DPPH Assay of Resveratrol

To measure the antioxidant property of resveratrol, DPPH assay was used. Five concentrations of resveratrol were tested and each sample of stock solution (2 µL of 100×) was added to 80 µL of 0.25 mM DPPH and 118 µL of 70% ethanol, to produce a final DPPH concentration of 0.1 mM. The mixture was vigorously shaken and left to stand for 30 min in the dark, and its absorbance was measured at 517 nm using an ELISA reader (TECAN, Salzburg, Austria).

### 4.3. ROS Scavenging Effects

Normal human melanocytes (NHMs) from teenager foreskin were cultured in modified MCDB 153 and incubated in a humidified incubator with 5% CO_2_ at 37 °C [33,34]. The cells were incubated after adding 50 µM of resveratrol. After 24 h, hydrogen peroxide (50 µM) was added and incubated for another 24 h. Then, cells were stained with H_2_DCF-DA (10 µM in PBS) for 30 min for fluorescent microscope examination.

### 4.4. Cell Culture and Viability

Normal human melanocytes were also cultured in modified MCDB 153 [33,34]. The cells were incubated another 2 h after adding CCK-8 solution (CK04, Dojindo, Rockville, MD, USA) to the cells. SpectraMax Plus Microplate Reader (Molecular Devices, Sunnyvale, CA, USA) was used to determine the quantity of water-soluble formazan produced by dehydrogenase activity of the cells [35].

### 4.5. Tyrosinase Activity and Melanin Amount

Cultured normal human melanocytes were incubated with resveratrol for three days. After washed with cold PBS, the cells were lysed with phosphate buffer (pH 6.8). Freezing-and-thawing broke down the cells, and the lysates were centrifuged at 10,000× *g* for 5 min. Each well of 96-well plate was filled with 90 µL of lysate and 10 µL L-DOPA, then incubated at 37 °C. ELISA was used to measure the absorbance at 475 nm every 10 min [35,36].

Melanin contents were measured. Briefly, cells were treated with resveratrol for 72 h. Cell pellets were then dissolved in 1mL of 1N NaOH at 100 °C for 30 min and centrifuged for 20 min at 16,000× *g*. Optical densities (OD) of the supernatants were measured at 400 nm using an ELISA reader. A standard synthetic melanin curve (0 to 300 mg/mL) was prepared in triplicate for each experiment.

Direct inhibition of tyrosinase induced by resveratrol was verified through a cell-free system. 20 µL of mushroom tyrosinase was added to 70 µL of phosphate buffer which contained resveratrol, followed by 10 µL of 10 mM l-DOPA. The absorbance at 475 nm was determined after incubating the mixture at 37 °C for 20 min [35].

### 4.6. Western Blot Analysis

The cells were placed in a cell lysis buffer (62.5 mM Tris–HCl (pH 6.8), 2% SDS, 5% β-mercaptoethanol, 2 mM phenylmethylsulfonyl fluoride, protease inhibitors (CompleteTM, Roche, Mannheim, Germany), 1 mM Na_3_VO_4_, 50 mM NaF, and 10 mM EDTA) [37]. SDS-PAGE separated 10 μg of protein per lane. The protein were blotted on the membranes and saturated with 5% dried milk. The blots were incubated with the primary antibodies at 1:1000 dilution, and then with horseradish peroxidase-conjugated secondary antibody. The enhanced chemiluminescence plus kit (Amersham International, Little Chalfont, UK) was used to detect the antibodies [35].

### 4.7. Fluorescence Microscopic Examination

80,000 normal human melanocytes were placed into 2-well chamber slides (Thermo Scientific Nunc, Rochester, NY, USA) and were treated with 20 μM of resveratrol. Culture of the cells was ceased at various time points during 24 h, followed by the fixation of the cells in 4% paraformaldehyde for 10 min. The cells were processed with 0.2% Triton X-100, 5% normal goat serum, and then incubated with the primary antibody. Alexa Fluo488^®^ goat anti-rabbit IgG (A11008, Molecular Probes^®^, Invitrogen, Carlsbad, CA, USA) at 1:1000 dilution was used to detect fluorescence [35].

### 4.8. Statistics

Statistical analyses were conducted using Microsoft Excel (Microsoft Corporation, Redmond, WA, USA) and IBM SPSS Statistics 22 (IBM Corporation, Armonk, NY, USA). Student’s *t*-test was used to measure differences among groups. *p* > 0.05 was considered to indicate statistical significance.

## 5. Conclusions

Our study verified that resveratrol could inhibit melanogenesis through the downregulation of MITF and tyrosinase through ERK pathway. In addition, we also confirmed that resveratrol was not a good direct inhibitor of tyrosinase in normal human melanocytes. Signaling pathway was analyzed and our findings clearly demonstrated that resveratrol may inhibit melanogenesis through the activation of FOXO3a but not by the activation of SIRT1. Although SIRT1 activation by resveratrol is a well-known mechanism of antiaging effects, our results suggested that SIRT1 is not involved in depigmenting effects of resveratrol.

## Supplementary Materials

Supplementary materials can be found at www.mdpi.com/1422-0067/18/6/1213/s1.

## Abbreviations

MITF	Microphthalmia-associated transcription factor
SIRT1	Sirtuin-1
FOXO	Forkhead box O
UV	Ultraviolet
IL	Interleukin
HGF	Hepatocyte growth factor
ET	Endothelin
KGF	Keratinocyte growth factor
SCF	Stem cell factor
bFGF	Basic fibroblast growth factor
α-MSH	α-melanocyte-stimulating hormone
ROS	Reactive oxygen species
AMPK	AMP-activated protein kinase
IGF	Insulin growth factor
l-DOPA	l-3,4-dihydroxyphenylalanine
ERK	Extracellular signal-regulate kinase
GAPDH	Glyceraldehyde 3-phosphate dehydrogenase
