# Supplementary material for: Depigmenting Effect of Resveratrol Is Dependent on FOXO3a Activation without SIRT1 Activation
Source: Int J Mol Sci. 2017 Jun 7;18(6):1213. doi: 10.3390/ijms18061213 (PMC5486036; doi:10.3390/ijms18061213)
Supplement: Supplementary file 1 [file ijms-18-01213-s001.pdf]

Supplementary Material: Depigmenting Effect of Resveratrol Is Dependent on FOXO3a Activation without SIRT1 Activation

Soon-Hyo Kwon, Hye-Ryung Choi, Youn-A Kang and Kyoung-Chan Park

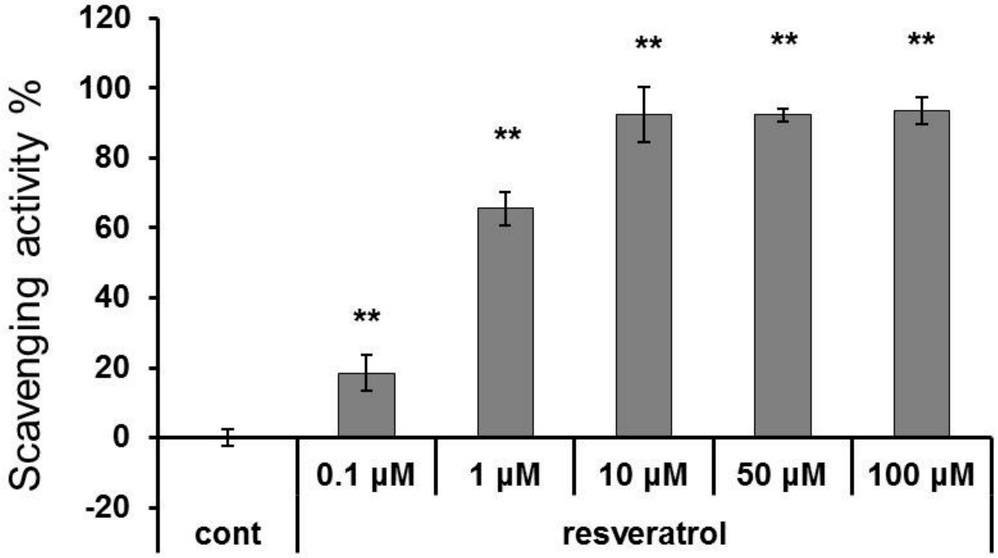

Figure S1. DPPH assay was performed in resveratrol at five concentrations (0.1µM to 100µM). Each sample of stock solution (2µl of 100X) was added to 80µl of 0.25 mM DPPH and 118µl of 70% ethanol, to produce a final DPPH concentration of 0.1 mM. The mixture was vigorously shaken, and its absorbance was measured at 517 nm using an ELISA reader.

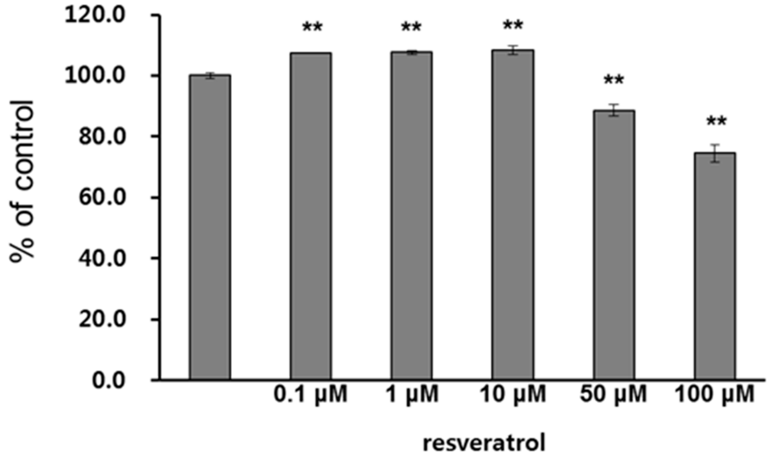

Figure S2. Cytotoxicity was performed at five different concentrations (0.1µM to 100µM). Normal human melanocytes were cultured and incubated with CCK-8 solution. The amount of water-soluble formazan generated by the activity of dehydrogenase was measured by an optical density at 450 nm using an ELISA reader.

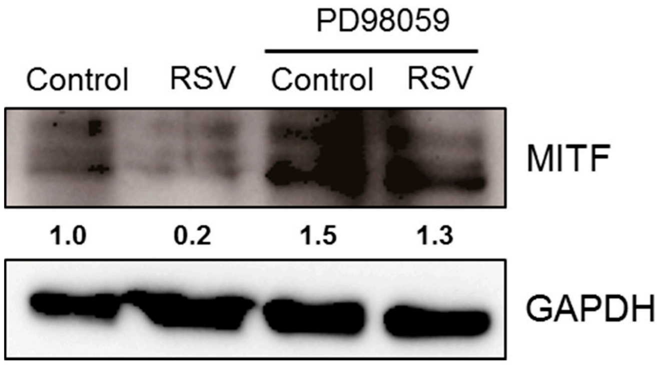

Figure S3. Western blotting was performed after PD98059 and resveratrol (50uM) treatment.
